# Antimicrobial activity of new green-functionalized oxazoline-based oligomers against clinical isolates

**DOI:** 10.1186/s40064-015-1166-5

**Published:** 2015-07-28

**Authors:** Celso Martins, Vanessa G Correia, Ana Aguiar-Ricardo, Ângela Cunha, Maria Guilhermina M Moutinho

**Affiliations:** CiiEM-Centro de Investigação Interdisciplinar Egas Moniz, Instituto Superior de Ciências da Saúde Egas Moniz Quinta da Granja, Campus Universitário, 2829-511 Caparica, Portugal; Departamento de Biologia & CESAM, Universidade de Aveiro, Campus Universitário de Santiago, 3810-193 Aveiro, Portugal; REQUIMTE, Departamento de Química, Faculdade de Ciências e Tecnologia, Universidade Nova de Lisboa, 2829-516 Caparica, Portugal

**Keywords:** Antimicrobial oxazolines, Opportunistic pathogens, MIC, Viability assay, Clinical isolates

## Abstract

**Background:**

The search for new antimicrobial compounds able to overcome the global issue of microbial resistance to antibiotics is a priority worldwide. Moreover, several commensal microorganisms have been increasingly associated to opportunistic microbial infections. Having previously disclosed the green synthesis and preliminary characterization of the oligomers [linear oligo(ethylenimine) hydrochloride and oligo(2-methyl-2-oxazoline) quaternized with *N,N*-dimethyldodecylamine] we herein report on the screening of these oligomers against a battery of 69 clinical isolates of *Aerococcus* spp., *Candida* spp., *Staphylococcus* spp. and *Streptococcus* spp.

**Findings:**

The isolates’ susceptibility to both oligomers was evaluated by determining their minimal inhibitory concentration (MIC) and the biocidal effectiveness of each compound was further confirmed through spectrophotometric measurements and fluorescence microscopy. The MIC values of the 69 isolates were highly variable, yet favourably comparable with those of other antimicrobial polymers. The viability assays resulted in 100% of microbial killing rate after only 5 min, highlighting the promising antimicrobial action of these oligomers.

**Conclusions:**

Though further studies are required, evidence suggests that a strong effort should be done in order to confirm these compounds as valid alternatives for several clinical applications. This is reinforced by their well described biocompatibility with human tissues and by their proposed mechanism of action which difficult the development of microbial resistance to these compounds.

**Electronic supplementary material:**

The online version of this article (doi:10.1186/s40064-015-1166-5) contains supplementary material, which is available to authorized users.

## Findings

### Oxazoline-based polymers

Microbial resistance to antibiotics is recognized as a major public health issue that urges rapid and effective resolution (Alanis 2005). This triggered the increasing attention that has been given to the search for alternative antimicrobial agents with higher effectiveness and with less risk for the development of similar resistance in a medium/long term (Aiello and Larson 2003; Hoogenboom 2009; Kenawy et al. 2007). In this context, polymeric antimicrobial materials have been recently considered as valuable alternatives to conventional antibiotics (especially as a preventive barrier for topical application and as coatings for critical surfaces), leading several authors to revisit poly(oxazoline)s potential after several decades of hiatus on their research (de la Rosa 2013; Kenawy et al. 2007; Hoogenboom 2009). The structural and functional characteristics of polymeric oxazolines, namely chemical stability, reduced toxicity, non-volatility properties, restricted permeation through the skin and potential as antimicrobial agents, turns them as promising alternatives to other well-known polymers already used in biomedical applications, such as e.g. polyethylene glycol (PEG) (de la Rosa 2013).

Even though the mechanism of action of these polymers remains unknown, it is thought to be identical to that of poly(peptides) (Waschinski et al. 2008). In a brief overview, it is hypothesised that poly(peptides) attach to the bacterial surface and cross the outer membrane of Gram-negative cells, or the thick layer of Gram-positive cells, by a self-promoted uptake to reach the anionic surface of the cytoplasmic membrane (Hancock and Sahl 2006). Finally, peptides disrupt the cell membrane by forming pores (barrel-stave model or toroidal-pore model) or by accumulating on the bilayer surface, covering it, dissolving the membrane in a detergent-like mode (carpet model) (Brogden 2005). Thus, poly(oxazoline)s are thought to present selectivity to negatively charged microbial cell envelopes relatively to the neutral mammalian cytoplasmic membranes, as occurs in poly(peptides) (Hancock and Sahl 2006). Consequently, poly(oxazoline)s are believed to be less susceptible to microbial resistance when compared to conventional antibiotics because it would imply a complete remodelling of the structure of the cellular membrane (Zasloff 2007).

### Green functionalized oligomers

An innovative green methodology (supercritical carbon dioxide technology) has been recently used by Aguiar-Ricardo and collaborators, aiming the development of sustainable and environmental friendly approaches to synthesize promising new functionalized oligo(oxazolines) (Correia et al. 2011; de Macedo et al. 2007). In previous work, the synthesis, chemical characterization, cytotoxicity and preliminary investigation of their potential as antimicrobial agents against the reference strains *Escherichia coli* AB1157 and *Staphylococcus aureus* NCTC8325-4 were undertaken (Correia et al. 2011). From a large set of preliminarily tested compounds, the two most promising were selected to be further investigated as antimicrobial agents against clinical isolates: linear oligo(ethylenimine) hydrochloride (hereafter defined as LOEI) and oligo(2-methyl-2-oxazoline) quaternized with *N,N*-dimethyldodecylamine (hereafter defined as OMETOX-DDA). These two oxazoline-based oligomers were obtained by cationic ring opening polymerization (CROP) in supercritical carbon dioxide which presents several advantages comparatively to conventional synthetic routes using organic solvents (see Supplementary Information for details on the synthesis and chemical characterization of the oligomers) (Correia et al. 2011). Among them, oligomers intrinsic blue fluorescence (Bonifácio et al. 2012), well-defined degrees of polymerization (generally are small polymers, oligomers), low polydispersity, high purity avoiding extensive purification in the end of the procedure are very important features when envisaging biomedical applications as, for example, 3D structures modification to confer antimicrobial and self-desinfection properties (Correia et al. 2013). This work acts as a follow-up study of Correia et al. (2011), expanded the antimicrobial efficiency of the selected oligomers against clinical isolates. Therefore, a susceptibility testing program against a battery of clinical isolates belonging to some relevant pathogenic taxa was conducted using well established methodologies (CLSI 2014), and fluorescence based viability tests were used on four of the isolates to confirm the biocidal effect of the compounds.

### Antimicrobial activity against clinical isolates

The strains tested in this study were previously isolated from clinical samples (Martins 2012) and are property of the Instituto Superior de Ciências da Saúde Egas Moniz. The selected strains comprise a total of 69 belonging to *Aerococcus* spp., *Candida* spp., *Staphylococcus* spp. and *Streptococcus* spp. These are Gram-positive aerobic and facultative anaerobic strains, tested due to the growing interest on studying the susceptibility of such microorganisms to alternative antimicrobials, since they are major colonizers of several human tissues (e.g. skin, mouth and respiratory tract) that frequently cause opportunistic infections (Alanis 2005; Miceli et al. 2011).

**Fig. 1:**
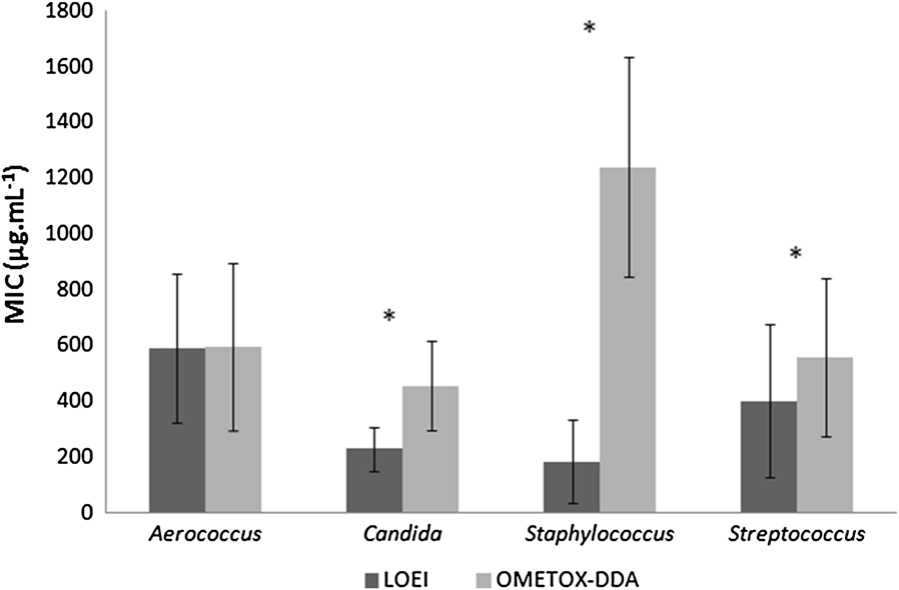
*Bar chart* containing the means of the MIC values (90% of inhibition) obtained for the four tested genus. Significant differences between the overall MICs of the two oligomers in each genus are marked with *asterisks*.

The MIC (90% of inhibition) values were obtained following the standard methodology implemented by the Clinical and Laboratory Standards Institute (CLSI 2014) using twofold dilutions from 6,240 to 6 µg mL^−1^ in Mueller-Hinton Broth (MHB) medium. Positive and negative controls were undertaken in parallel with the tests, consisting respectively in (1) MHB inoculated with the respective tested microorganism (no oligomer) and (2) three different dilutions of the oligomers in MHB medium and also only MHB to assure sterility (no inoculation in both negatives). Additionally, the full taxonomic details of each isolate, respective result on the MIC values and standard deviation are displayed in Additional file 1: Table S1, revealing high variability of MICs between and within taxa. An overview of the obtained MIC values is depicted in Fig. 1, which exposes important differences when comparing the means of both oligomers at the four taxa. *Candida* spp., *Staphylococcus* spp. and *Streptococcus* spp showed significant higher susceptibility to LOEI than to OMETOX-DDA (*p* value <0.05). *Aerococcus* spp. displayed similar means of MIC values to both oligomers. Importantly, LOEI shows an overall stronger inhibitory effect than OMETOX-DDA, except against *Aerococcus* spp. This variability in MIC values is not surprising when considering previous reports on the differences of susceptibility to drugs between different types of microorganisms, mostly related with their differences in the thickness of the peptidoglycan layer of the cell membrane and their cell wall structures, which are variable within different strains of the same species (Timofeeva and Kleshcheva 2011).

Following the results obtained in MIC determination one strain of *Aerococcus urinae* (facultative pathogen, colonizer of urinary tract), *Candida albicans* (representing pathogenic yeasts), *Staphylococcus aureus* (one of the most studied opportunistic pathogens, major colonizer of the skin and mucosa) and *Streptococcus mutans* (mutans streptococci, responsible for caries and oral infections) were submitted to viability assays using a concentration of fivefold the established MIC in MHB media for each of the tested strain (bolded in Additional file 1: Table S1). The media also contained propidium iodide (PI) labelling to allow measuring the fluorescence emissions along time, indicative of death by membrane disruption and hybridization of PI with nucleic acids. Aliquots were removed at 5 and at 30 min, washed with saline solution to remove any excess of propidium iodide and observed with a DM5500 B fluorescence microscope (Leica) using a N21 filter set, a 100× magnification objective and images were captured with an Andor Luca R EMCCD camera. Additionally, similar aliquots were also plated in solid media to confirm microbial death. Positive (standard cultivation in MHB without any oligomer but with PI labelling) and negative (MHB with no cells) controls were undertaken for all these procedures.

**Fig. 2:**
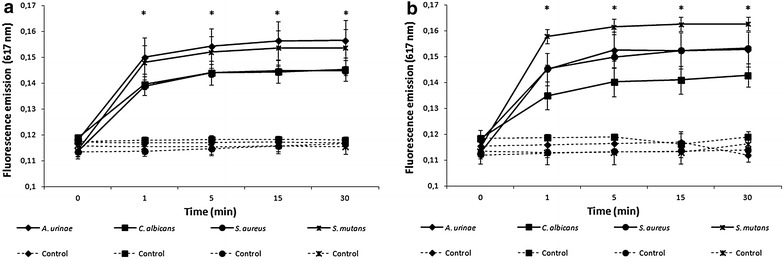
Plots of the fluorescence emission of four different clinical isolates when in contact with **a** LOEI and **b** OMETOX-DDA. Statistically significant differences relatively to the control were detected in fluorescence emission at all measured time points for all the tested strains (marked with *asterisks*), with the exception of time 0.

In Fig. 2 it is possible to observe significant increasing of fluorescence emission (*p* value <0.05) after one single minute of action and then a slight increase after 5 min followed by an overall stabilization of the emissions till the final time point at 30 min. The statistical analysis (Mann–Whitney and *t* tests) revealed that the increase on fluorescence emission was significant relatively to the positive controls during the whole experiment (*p* values <0.05).

**Fig. 3:**
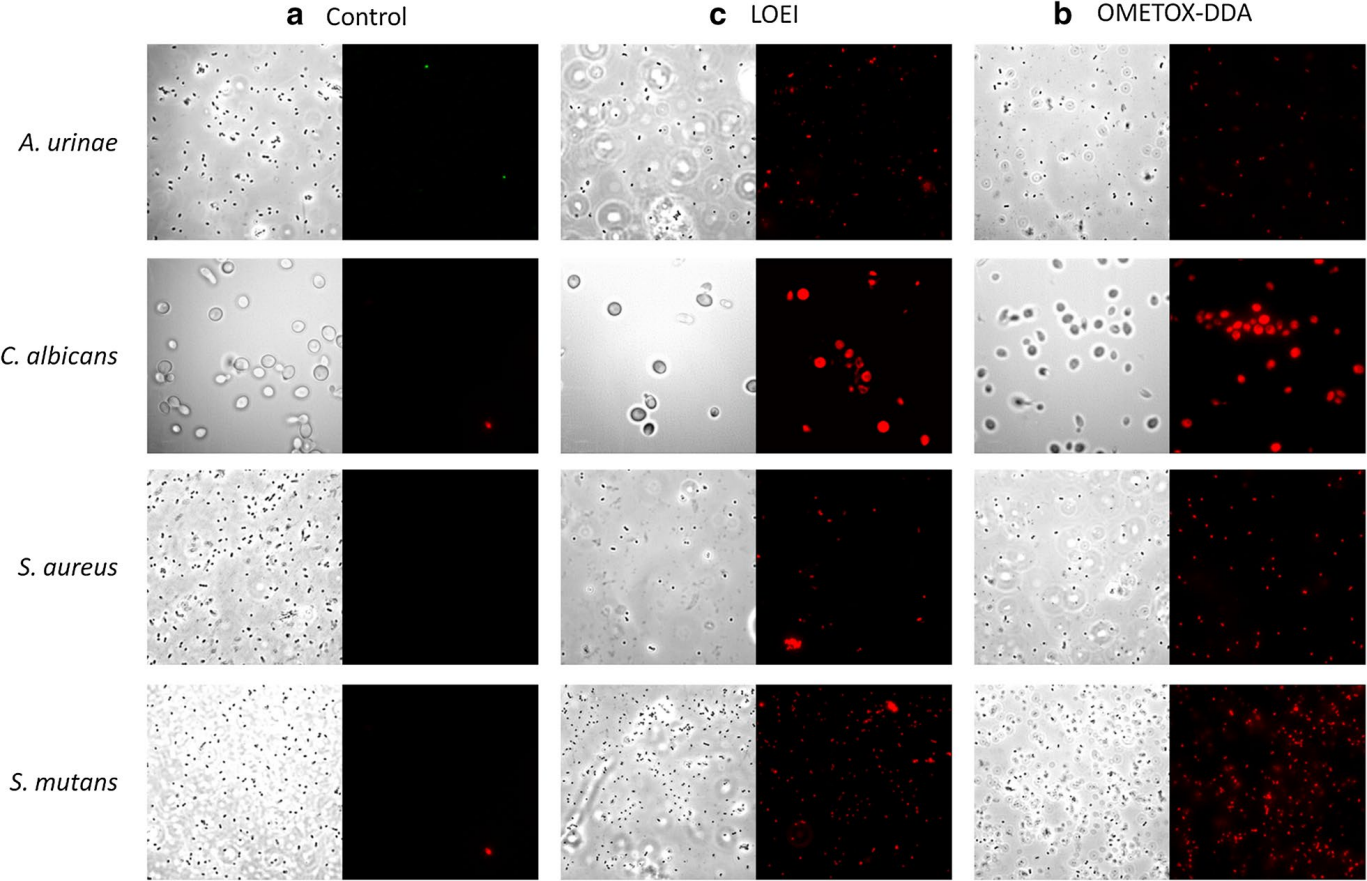
Illustrative micrographs of the fluorescence microscopy analysis exposing the microbiocidal activity of the tested compounds. For each compound **a** control, **b** LOEI and **c** OMETOX-DDA) the total community is depicted in the *left column* (contrast-phase microscopy) and the death cells are observable in *red* (propidium iodide) in the *right column*.

When comparing the results in the present work with the preliminary assays undertaken with reference strains (Correia et al. 2011), we noticed some interesting differences in the time of death. With reference strains the oligomer 5 (LOEI in this work) took *ca.* 75 min to kill *E. coli* and 4 h to effectively kill *S. aureus* cells contrasting with the 5 min necessary to achieve bacterial kill reported herein; however, in that work the oligomer 1b (OMETOX-DDA in this work) revealed similar results to those obtained in the present contribution, killing bacterial cells after 5 min of incubation. In Correia et al. (2011) the differences in time of action between the two oligomers were assumed to be a consequence of distinct mechanisms (described above). However the shorter killing times herein verified with LOEI suggests that the use of a concentration fivefold higher than the MIC values provides effective and quick killing of clinical isolates, contrasting with reference strains, illustrated by fluorescence microscopy micrographs (Fig. 3) and confirmed using plate inoculation after 5 and 30 min in contact with the two compounds, which retrieved no microbial growth in solid media, in contrast with the normal growth obtained for positive control. This emphasizes the potential of both oligomers as powerful biocidal agents against both yeasts and bacteria, which is probably related to the negatively charged cell wall of both groups of microorganisms (Ibeas et al. 2000).

Importantly, these compounds have previously shown selectivity towards microbial cells. Correia et al. observed that their bactericidal activity is much faster than their cytotoxic effect in mammalian cells, as the latest are still metabolically active after 42 h in the presence of 0.1 mg mL^−1^. In that work, metabolic decay was observed in mammalian cells after 48 h using concentrations around 10 mg mL^−1^ (Correia et al. 2011). Thus, both concentrations and time of exposure largely exceed the MIC values that they established for reference strains. Similar analysis can be inferred in this work having as basis the cytotoxicity tests undertaken by Correia et al. since that the kinetics of antibacterial activity are so fast (compared to the time it takes for mammalian toxicity to occur) that there is a therapeutic window for the application of these oligomers, even if the concentrations used during the viability assays were fivefold of the established MIC for each strain, thus approaching to the toxic level to mammalian cells (10 mg mL^−1^). This opens the door for future studies due to their slow onset of toxicity, still our data revealed that these oligomers will hardly become suitable for prolonged application and/or contact time.

Despite the fact that the used concentrations in this work may look extremely high, especially if compared with standard antibiotics, such comparison should be performed with chemicals presenting similar chemical properties and/or mechanism of action. Most of the conventionally used antibiotics act mostly as metabolic and/or protein synthesis inhibitors (e.g. ampicillin and rifampicin) and are commonly used for the treatment of systemic infections. Due to their mechanism of action, small size and molecular weight, the oligomers described herein might be comparable with other antimicrobial polymers, with conventional antimicrobial peptides (AMP’s) or even with new classes of rare and cryptic secondary metabolites, such as peptaibols (Duclohier 2010). A closer look to the literature reveals that several of these compounds reported antimicrobial activity at concentrations closer to 10 mg mL^−1^ such as e.g. protamine, a well known AMP (Aspedon and Groisman 1996). Such values are considerably higher than the ones considered in the present work, so we can state that when compared to similar chemicals with similar antimicrobial mechanisms, the oligomers studied herein present encouraging results (de la Rosa 2013).

Of relevance is also the sustainable and environmental friendly approach used for the synthesis of these compounds constituting an important advance due to the increasing concerns regarding the environmental safety during the production of new synthetic antimicrobials (Martinez 2009).

The evaluation of the biocidal potential of these two functionalized oligomers against a wide range of clinical isolates demonstrated therapeutic potential. Nevertheless, further in vitro preclinical testing is needed before these compounds can be scaled up to multi-gram levels for in vivo evaluation. We believe that investigations exploring the action of these oligomers against clinical isolates of Gram-negative, enteric and anaerobic microorganisms should also be soon conducted.

This work confirms the promising potential of both oxazoline based oligomers against clinical isolates, and emphasises that the development and improvement of this class of materials may help solving some current issues in terms of epidemiology (de la Rosa 2013). The chemical and antimicrobial properties of these oligomers open the door for future topical and antimicrobial coating applications (de la Rosa 2013), strategies in line with a preventive philosophy as the first line of defence against microbial infections.

## Additional file

Additional file 1: Additional file is available online. The full synthesis and chemical characterization of the oligomers is shown in Sect. 1. Additionally, the full numbered list of the clinical isolates and respective MIC values plus the respective standard deviations are displayed in Table S1.
