# Supplementary material for: Antimicrobial activity of new green-functionalized oxazoline-based oligomers against clinical isolates
Source: Springerplus. 2015 Jul 28;4:382. doi: 10.1186/s40064-015-1166-5 (PMC4516143; doi:10.1186/s40064-015-1166-5)
Supplement: Additional file 1: — Additional file is available online. The full synthesis and chemical characterization of the oligomers is shown in Sect. 1. Additionally, the full numbered list of the clinical isolates and respective MIC values plus the respective standard deviations are displayed in Table S1. [file 40064_2015_1166_MOESM1_ESM.docx]

Supplementary Information

**Antimicrobial activity of new green-functionalized oxazoline-based oligomers against clinical isolates**

Celso Martins^1,2^, Vanessa G. Correia^3^, Ana Aguiar-Ricardo^3^, Ângela Cunha^1^, Guilhermina M. Moutinho^2*^

^1^ Departamento de Biologia & CESAM, Universidade de Aveiro, Campus Universitário de Santiago, 3810-193 Aveiro, Portugal

^2^ CiiEM – Centro de Investigação Interdisciplinar Egas Moniz, Instituto Superior de Ciências da Saúde Egas Moniz Quinta da Granja, Campus Universitário, 2829-511 Caparica, Portugal

^3^ REQUIMTE, Departamento de Química, Faculdade de Ciências e Tecnologia, Universidade Nova de Lisboa, 2829-516 Caparica, Portugal

*corresponding author: mgm.moutinho@gmail.com

**Synthesis of antimicrobial Oligo(2-oxazoline)s**

Please notice that both polymers were synthesized as previously reported in the reference (Correia et al. 2011) of the main text.

**Materials**

The monomers 2-methyl-2-oxazoline (MeOx) and the initiator boron trifluoride diethyl etherate (BF_3_.OEt_2_) were purchased from Sigma-Aldrich. *N,N*-dimethyldodecylamine was purchased from Fluka. All the reagents were used without further purification. Carbon dioxide was supplied by Air Liquide with a purity of 99.998%.

**Synthesis of *Living* Oligo(2-methyl-2-oxazoline).** The polymerization was carried out in a stainless-steel reactor equipped with two aligned sapphire windows stamped in both tops with Teflon o-rings. 2-methyl-2-oxazoline was used as the monomer and boron trifluoride etherate (BF_3_.Et_2_O) was used as the initiator. The monomer/initiator ratio used to each polymerization was [M]/[I]=15. The reactor cell was charged with the monomer, the initiator, a magnetic stirring bar, and then immersed in a thermostatized water bath. Carbon dioxide was introduced in the reactor in order to achieve the desired reaction pressure (from 16 to 20 MPa). After 20 hours of reaction, the pressure was slowly released and the reactor led to reach room temperature. Inside the reactor, a solid was obtained.

**End-capping of *Living* Oligo(2-methyl-2-oxazoline).** At the end of the polymerization, the *living* oligomer was end-capped with *N,N*-dimethyldodecylamine. The functionalization was achieved by the addition of a tenfold excess of the terminating agent relatively to the initiator. The mixture was kept at 70 ºC under stirring for 24 hours. Powder oligomers were washed with diethyl ether and dried under vacuum.

**Preparation of Linear Oligo(ethylenimine) Hydrochloride.** Oligo(2-methyl-2-oxazoline) was submitted to hydrolysis in a 5M HCl aqueous solution under reflux for 9 hours. After this period the polymer precipitated as the hydrochloride salt. The mixture was then filtered off, washed with acetone and dried under vacuum and linear oligo(ethylenimine) hydrochloride was obtained as a white solid.

**Chemical characterization of antimicrobial Oligo(2-oxazoline)s**

**OMETOX-DDA**

Yellow solid. Water soluble. Hygroscopic. Yield: 68%. FTIR (KBr) ν_máx_ (cm^-1^): 1738 (NCO_2_), 1634 (NCOMe). ^1^H-NMR (400 MHz, CDCl_3_): δ - 0.87 (t, 3H, *J*= 6.3 Hz, H-3), 1.24 (bs, 16H, H-4), 1.71 (bs, 2H, H-5), 2.13 (bs, 3H, H-2), 3.01 (t, 2H, *J*= 8.0 Hz, H-6), 3.15 (bs, 2H, H-7), 3.44 (bs, 10H, overlapped signals, H-1 + H-8). ^13^C-NMR (100 MHz, CDCl_3_): δ - 14.10, 21.15, 22.65, 24.48, 26.44, 29.80, 29.35, 29.56, 31.86, 43.49, 44.95, 47.05, 47.84, 58.63, 170.88, 171.62. *M*_n_= 1237 g.mol^-1^ (n*=* 12) by NMR. MALDI-TOF (after hydrolysis): *M*_n_= 505 g.mol^-1^.

**LOEI**

White solid. Water soluble. Yield: 74%. FTIR (KBr) ν_máx_ (cm^-1^): 3420 (NH). ^1^H-NMR (400 MHz, D_2_O): δ - 3.48 (s, 4H, H-1). ^13^C-NMR (100 MHz, D_2_O): δ - 43.83. MALDI-TOF: *M*_n_= 491 g.mol^-1^ (n= 11)

**Table 1 –** Full numbered list of the clinical isolates, alphabetically ordered according to their taxonomic classification. MIC values are displayed for each individual strain. The strains used in death assays are bolded.

| **Strain** | **Species** | **MIC LOEI** | | **MIC OMETOX-DDA** | |  | **Strain** | **Species** | **MIC LOEI** | | **MIC OMETOX-DDA** | |
| --- | --- | --- | --- | --- | --- | --- | --- | --- | --- | --- | --- | --- |
|  |  | **(µg.mL^-1^)** | **std. dev.** | **(µg.mL^-1^)** | **std. dev.** |  |  |  | **(µg.mL^-1^)** | **std. dev.** | **(µg.mL^-1^)** | **std. dev.** |
| 1 | *Aerococcus urinae* | 780 | 225 | 780 | 0 |  | 36 | *Streptococcus intermedius* | 780 | 225 | 780 | 225 |
| 2 | *Aerococcus urinae* | 390 | 113 | 780 | 225 |  | 37 | *Streptococcus intermedius* | 390 | 113 | 780 | 225 |
| 3 | *Aerococcus urinae* | 780 | 225 | 780 | 225 |  | 38 | *Streptococcus mitis* | 780 | 0 | 195 | 56 |
| **4** | ***Aerococcus urinae*** | **780** | **0** | **780** | **0** |  | 39 | *Streptococcus mitis* | 48 | 29 | 780 | 225 |
| 5 | *Aerococcus urinae* | 390 | 0 | 98 | 29 |  | 40 | *Streptococcus mitis* | 780 | 225 | 780 | 225 |
| 6 | *Aerococcus urinae* | 780 | 225 | 780 | 225 |  | 41 | *Streptococcus mitis* | 780 | 225 | 780 | 0 |
| 7 | *Aerococcus urinae* | 390 | 113 | 780 | 225 |  | 42 | *Streptococcus mitis* | 780 | 225 | 390 | 113 |
| 8 | *Aerococcus urinae* | 48 | 29 | 195 | 56 |  | 43 | *Streptococcus mitis* | 195 | 0 | 195 | 56 |
| 9 | *Aerococcus urinae* | 780 | 225 | 780 | 225 |  | 44 | *Streptococcus mitis* | 195 | 56 | 98 | 29 |
| 10 | *Aerococcus urinae* | 780 | 225 | 195 | 56 |  | 45 | *Streptococcus mitis* | 780 | 225 | 98 | 29 |
| **11** | ***Candida albicans*** | **390** | **0** | **390** | **113** |  | 46 | *Streptococcus mutans* | 98 | 29 | 48 | 29 |
| 12 | *Candida albicans* | 195 | 56 | 780 | 225 |  | 47 | *Streptococcus mutans* | 98 | 29 | 390 | 113 |
| 13 | *Candida albicans* | 195 | 56 | 390 | 0 |  | **48** | ***Streptococcus mutans*** | **98** | 29 | **780** | 0 |
| 14 | *Candida albicans* | 195 | 56 | 390 | 113 |  | 49 | *Streptococcus mutans* | 98 | 29 | 780 | 225 |
| 15 | *Candida albicans* | 195 | 0 | 390 | 113 |  | 50 | *Streptococcus oralis* | 780 | 225 | 780 | 0 |
| 16 | *Candida albicans* | 195 | 56 | 390 | 0 |  | 51 | *Streptococcus oralis* | 390 | 113 | 195 | 56 |
| 17 | *Staphylococcus aureus* | 195 | 56 | 1560 | 0 |  | 52 | *Streptococcus salivarius* | 390 | 113 | 780 | 0 |
| 18 | *Staphylococcus aureus* | 195 | 56 | 1560 | 450 |  | 53 | *Streptococcus salivarius* | 780 | 225 | 780 | 225 |
| 19 | *Staphylococcus aureus* | 195 | 56 | 1560 | 0 |  | 54 | *Streptococcus salivarius* | 390 | 0 | 780 | 0 |
| 20 | *Staphylococcus aureus* | 48 | 29 | 780 | 0 |  | 55 | *Streptococcus salivarius* | 390 | 113 | 780 | 225 |
| 21 | *Staphylococcus aureus* | 48 | 29 | 780 | 225 |  | 56 | *Streptococcus salivarius* | 780 | 225 | 780 | 225 |
| 22 | *Staphylococcus aureus* | 390 | 0 | 780 | 225 |  | 57 | *Streptococcus salivarius* | 390 | 113 | 390 | 0 |
| **23** | ***Staphylococcus aureus*** | **390** | **0** | **1560** | **0** |  | 58 | *Streptococcus salivarius* | 390 | 0 | 780 | 225 |
| 24 | *Staphylococcus aureus* | 390 | 113 | 1560 | 0 |  | 59 | *Streptococcus salivarius* | 780 | 225 | 195 | 56 |
| 25 | *Staphylococcus aureus* | 195 | 56 | 780 | 225 |  | 60 | *Streptococcus salivarius* | 195 | 56 | 195 | 56 |
| 26 | *Staphylococcus capitis* | 48 | 29 | 780 | 225 |  | 61 | *Streptococcus salivarius* | 390 | 113 | 780 | 0 |
| 27 | *Staphylococcus capitis* | 48 | 29 | 780 | 0 |  | 62 | *Streptococcus salivarius* | 98 | 29 | 780 | 225 |
| 28 | *Staphylococcus epidermidis* | 48 | 0 | 780 | 0 |  | 63 | *Streptococcus salivarius* | 48 | 29 | 390 | 113 |
| 29 | *Staphylococcus epidermidis* | 48 | 29 | 1560 | 450 |  | 64 | *Streptococcus salivarius* | 195 | 56 | 780 | 0 |
| 30 | *Staphylococcus epidermidis* | 48 | 29 | 1560 | 450 |  | 65 | *Streptococcus sanguinis* | 195 | 56 | 780 | 225 |
| 31 | *Staphylococcus epidermidis* | 48 | 0 | 1560 | 450 |  | 66 | *Streptococcus sanguinis* | 780 | 0 | 780 | 225 |
| 32 | *Staphylococcus xylosus* | 390 | 0 | 1560 | 0 |  | 67 | *Streptococcus sanguinis* | 195 | 56 | 780 | 225 |
| 33 | *Staphylococcus xylosus* | 390 | 113 | 1560 | 450 |  | 68 | *Streptococcus suis* | 390 | 113 | 390 | 113 |
| 34 | *Streptococcus bovis* | 195 | 56 | 98 | 29 |  | 69 | *Streptococcus suis* | 195 | 56 | 780 | 0 |
| 35 | *Streptococcus bovis* | 195 | 56 | 390 | 113 |  |  |  |  |  |  |  |
